# The reliability and validity of repeat power ability assessments and measurement indices in loaded vertical jumps

**DOI:** 10.7717/peerj.15553

**Published:** 2023-06-20

**Authors:** Alex O. Natera, Dale W. Chapman, Neil D. Chapman, Justin W.L. Keogh

**Affiliations:** 1 Faculty of Health Sciences and Medicine, Bond University, Gold Coast, Queensland, Australia; 2 Sport Science, New South Wales Institute of Sport, Sydney, New South Wales, Australia; 3 Curtin School of Allied Health, Curtin University, Perth, Western Australia, Australia; 4 Sports Performance Research Centre New Zealand, Auckland University of Technology, Auckland, New Zealand; 5 Cluster for Health Improvement, Faculty of Science, Health, Education and Engineering, University of the Sunshine Coast, Sunshine Coast, Queensland, Australia; 6 Kasturba Medical College, Manipal Academy of Higher Education, Manipal, Karnataka, India

**Keywords:** Power output, Vertical jump, Repeat power ability, Fatigue index, Percent decrement, Power endurance, Ballistic, Anaerobic capacity, High intensity, Loaded jumps

## Abstract

**Background:**

Repeat power ability (RPA) assessments are a valuable evaluation of an athlete’s ability to repeatedly perform high intensity movements. Establishing the most reliable and valid loaded jump RPA assessment and method to quantify RPA has yet to be determined. This study aimed to compare the reliability and validity of an RPA assessment performed with loaded squat jumps (SJ) or countermovement jumps (CMJ) using force-time derived mean and peak power output.

**Materials and Methods:**

RPA was quantified using calculations of average power output, a fatigue index and a percent decrement score for all repetitions and with the first and last repetitions removed. Validity was established by comparing to a 30 second Bosco repeated jump test (30BJT). Eleven well-trained male field hockey players performed one set of 20 repetitions of both SJs (20SJ) and CMJs (20CMJ) on separate occasions using a 30% one repetition maximum half squat load. These assessments were repeated 7 days apart to establish inter-test reliability. On a separate occasion, each participant performed the 30BJT.

**Results:**

The reliability of average peak power for 20SJ and 20CMJ was acceptable (CV < 5%; ICC > 0.9), while average mean power reliability for 20CMJ (CV < 5%; ICC > 0.9) was better than 20SJ (CV > 5%; ICC > 0.8). Percent decrement of 20CMJ peak power, with the first and final jump removed from the percent decrement calculation (PD%CMJ_peak18_), was the most reliable measurement of power output decline (CV < 5 %; ICC > 0.8). Average mean and peak power for both RPA protocols had moderate to strong correlations with 30BJT average mean and peak power (r = 0.5–0.8; *p*< 0.05–0.01). No RPA measurements of power decline were significantly related to BJT measurements of power decline.

**Conclusions:**

These findings indicate that PD%CMJ_peak18_ is the most reliable measure of RPA power decline. The lack of relationship between power decline in the loaded RPA and the 30BJT assessment suggest that each assessment may be measuring a different physical quality. These results provide sport science practitioners with additional methods to assess RPA and provide useful information on the reliability and validity of these outcome measures. Additional research needs to be performed to examine the reliability and validity of the novel RPA assessments in other athletic populations and to determine the sensitivity of these measurements to training and injury.

## Introduction

The ability to perform multiple high intensity efforts, termed repeat power ability (RPA) is a key component of many team sports (Mohr, Krustrup & Bangsbo, 2003; Spencer et al., 2004; Austin, Gabbett & Jenkins, 2011). RPA can be defined as the capacity to repeatedly produce maximal muscular efforts against external resistances (Natera, Cardinale & Keogh, 2020). A range of metrics have been used to describe and quantify RPA performance, with mean power output the most common (Patterson, Platzer & Raschner, 2019; Apanukul, Suwannathada & Intiraporn, 2015; Baker & Newton, 2007). Despite mean power output being the most common measure of RPA, Alemany et al. (2005) reported peak power output to be more reliable than mean power output (ICCs between 0.94–0.96 *versus* 0.89–0.96 and CVs 3.2% *vs* 4.4% respectively) in a 1 × 30 repetition continuous rebound jump protocol, where upon landing the subjects immediately descended to a self-selected squat depth before commencing the upward jumping movement. Ultimately, there is still relative uncertainty regarding what might be the most appropriate human movement *e.g.*, jump type as well as ways to calculate the outputs *e.g.*, fatigue index, percent decrement and if such outputs should be calculated using mean or peak power values.

Although it is likely that a combination of neural, metabolic, and mechanical factors influence RPA, there is a paucity of research explaining and investigating the performance characteristics of RPA. In contrast there is a plethora of evidence examining anaerobic capacity and the role of the phosphagen and fast glycolytic system on repeated sprint ability (Girard, Mendez-Villanueva & Bishop, 2011; Granatelli et al., 2014; Soares-Caldeira et al., 2014). Although the Wingate anaerobic test is often used to measure anaerobic power and capacity, it is suggested that different mechanisms of maximal power and fatigue are used when compared to assessments of RPA (Fry et al., 2014). The acyclical repetitions used in RPA assessments may cause more of a reliance on the phosphagen system in comparison to the cyclical Wingate anaerobic test (Fry et al., 2014). The external loads used along with the stretch-shortening cycle (SSC) actions and the landing forces experienced in some RPA jump assessments *e.g.*, 60 s Bosco Jump Test (60BJT) may also cause a greater stress on the neuromuscular system in comparison to the Wingate anaerobic test (Sands et al., 2004; Fry et al., 2014; Natera, Cardinale & Keogh, 2020).

The 60BJT was the first RPA assessment developed to better replicate the ballistic characteristics and SSC actions seen in many sports (Bosco et al., 1983). The 60BJT is performed with bodyweight (no additional resistance) and has been used to assess both anaerobic power and anaerobic capacity (Bosco et al., 1983). The 60BJT has also been used to differentiate between athletic cohorts and estimate muscle fiber type composition (Bosco et al., 1983; Bosco, Luhtanen & Komi, 1983; Sands et al., 2004). Notwithstanding the support for the use of the 60BJT, abbreviated forms of the 60BJT such as the 30 s Bosco Jump Test (30BJT), have been utilised in recent years to mediate excessive fatigue during testing which can negatively interfere with subsequent elements of training (Dal Pupo et al., 2014; Pupo et al., 2013; Trinkūnas et al., 2011). The 30BJT has been reported as a reliable and valid assessment of anaerobic power and capacity, particularly in athletes who require the use of stored elastic energy for performance purposes (Dal Pupo et al., 2014; Pupo et al., 2013; Trinkūnas et al., 2011).

One criticism of RPA assessments such as the 30BJT and 60BJT, is that they do not utilise external loading, and as such they may not adequately represent the external load experienced in many sports (Natera, Cardinale & Keogh, 2020). As such, unloaded RPA assessments may not provide sufficient stress to the neuromuscular and cardiorespiratory system to replicate loading strategies typically used to improve power output (Natera, Cardinale & Keogh, 2020). The Kansas Squat Test is an RPA assessment that was developed to address this lack of loading, involving 15 repetitions of speed squats with a system mass back squat load of 70% 1RM. Reliability and validity of the Kansas Squat Test was established against the Wingate anaerobic test (ICCs = 0.754 to 0.937 and *r* = 0.775 respectively) (Fry et al., 2014), but not the 60BJT or 30BJT. However, it is important to consider that speed variations of traditional strength exercises, like the speed squat used by Fry et al. (2014), can have up to 40% of the concentric portion of the lift used to decelerate the barbell (Elliott, Wilson & Kerr, 1989).

Although Fry et al. (2014) is the only group to investigate the validity of an RPA assessment, other studies using loaded CMJs have reported their protocol’s reliability. RPA assessments using loaded CMJs have reported ICCs of between 0.73 to 0.97 and 0.881 to 0.987, respectively (Alemany et al., 2005; Patterson, Raschner & Platzer, 2014). However, these RPA protocols differed considerably with Alemany et al. (2005) using a Smith machine for 1 set of 30 repetitions and Patterson, Raschner & Platzer (2014) using a free barbell for a 2.5-minute protocol. Further differences between protocols were seen in the loads and measurement device used (rotary encoder *vs.* force plate respectively). Further differences between protocols were seen in the loads and measurement device used (rotary encoder *vs.* force plate respectively).

Patterson, Raschner & Platzer (2014) sought to establish more control over their CMJ protocol by standardising the time between jump and the depth of countermovement. Only Patterson, Raschner & Platzer (2014) used a maximal power reference value, to minimise possible pacing strategies. Although the Alemany et al. (2005) protocol was reliable, an accurate measure of RPA might not have been established without controlling these factors. For example, allowing a self-selected countermovement depth may have resulted in participants changing their strategy and utilising a shallower countermovement as they fatigued. This may have artificially inflated power output by decreasing the time to perform work. Unlike Patterson, Raschner & Platzer (2014), Alemany et al. (2005) did quantify load relative to strength levels, and as a result of using a Smith machine, the requirement to control the vertical tracking of the bar was lessened.

Ballistic movements may better replicate many of the actions performed in sports where acceleration throughout the entire range of motion is important. As such RPA assessments performed with ballistic exercises, like loaded CMJs, involve greater acceleration of the barbell throughout the entire movement (Newton et al., 1996). However, with changes in countermovement strategy observed as a result of fatigue, controlling for technical influence in the countermovement strategy might be provided by using a squat jump (SJ) (Cormack et al., 2008). To date no studies have investigated the SJ as a potential ballistic exercise for RPA assessments. With a SJ performed from a static position, the countermovement strategies used in a CMJ are largely avoided. However, it is yet to be determined whether under fatigue, a SJ can be truly performed without some form of a small amplitude countermovement, particularly when maximal power output for each repetition is the objective.

The method of calculation of power decline is a further source of discrepancy in the RPA literature. To quantify the change in power output across multiple repetitions in RPA, Baker & Newton (2007) and Fry et al. (2014) calculated the percent difference in mean power output between the final repetition and the repetition with the highest mean power output. Patterson, Platzer & Raschner (2019) quantified the change in power output as the percentage difference between the jump that registered the highest power output with the average power output of the final 12 jumps in the assessment. In an investigation of four different approaches to quantify the ability to repeatedly produce high intensity efforts, the percent decrement calculation was found to be the most valid and reliable method of quantifying fatigue (ICCs between 0.81–0.83) (Glaister et al., 2004). The percent decrement calculation identifies the percent difference between the total sum and the ideal sum. Currently, no RPA assessments utilise the percent decrement method to quantify RPA.

In repeated high intensity efforts, where the overall repetitions to be performed are known by the participant, there may be a risk of pacing to mitigate the earlier onset of fatigue (Billaut et al., 2011). Participants may consciously or unconsciously use pacing during RPA assessments, therefore it may be important to control for the most variable jumps within the assessment by removing them from the calculation of power decline. To our knowledge the removal of potentially highly variable jumps, that may occur at the very start and very end of an RPA assessment, have not been performed before to improve RPA assessment reliability.

The aim of this study was to: (1) compare the inter-test reliability of an RPA assessments consisting of loaded SJs and an RPA assessment consisting of loaded CMJs, using an average score, a fatigue index and a percent decrement score, for both mean and peak power output; and (2) assess the validity of the two RPA assessments and the different measurement indices against the 30BJT. We hypothesized that: (1) the CMJ protocol would be more reliable than the SJ protocol and that the peak power percent decrement score would be the most reliable measure of RPA; and (2) none of the measured RPA variables would be valid in comparison to the 30BJT.

## Materials & Methods

### Participants

Eleven well-trained male field hockey players (age 21.6  ± 2.4 years, body mass 78.2 kg  ± 6.8, stature 182.1  ± 5.3 cm) currently playing at state level competition volunteered to participate in this study. Each of the players had a minimum of three years resistance training experience and regularly trained with near maximal squats and loaded jumps. Each participant performed the investigation during pre-season and all training requirements were controlled and matched from one week to the next. A full explanation of the assessment procedures was given to each participant along with a weight training history questionnaire, to verify their eligibility for this study. Exclusion criteria for this study included participants with less than 3 years of resistance training experience and participants who were not currently performing near maximal squats and loaded jumps for at least 4 weeks leading up to the start of data collection. All participants were asked to complete and provide their informed consent document, and the investigation was approved by Bond University Human Research Ethics Committee (N00156).

### Study design

This study investigated the inter-test reliability of two different RPA assessment movements (SJ and CMJ) with a confirmation assessment to a criterion test (30BJT) using a counter balanced randomized test-retest design. Twenty repetitions of SJs (20SJ) and 20 repetitions of CMJs (20CMJ) with a 30% 1RM half squat load were performed using a Smith machine for the RPA assessments. Absolute and relative peak and mean power calculations for each repetition of both the 20SJ and 20CMJ and the 30BJT were used to quantify power decline.

### Procedures

**Figure 1:**
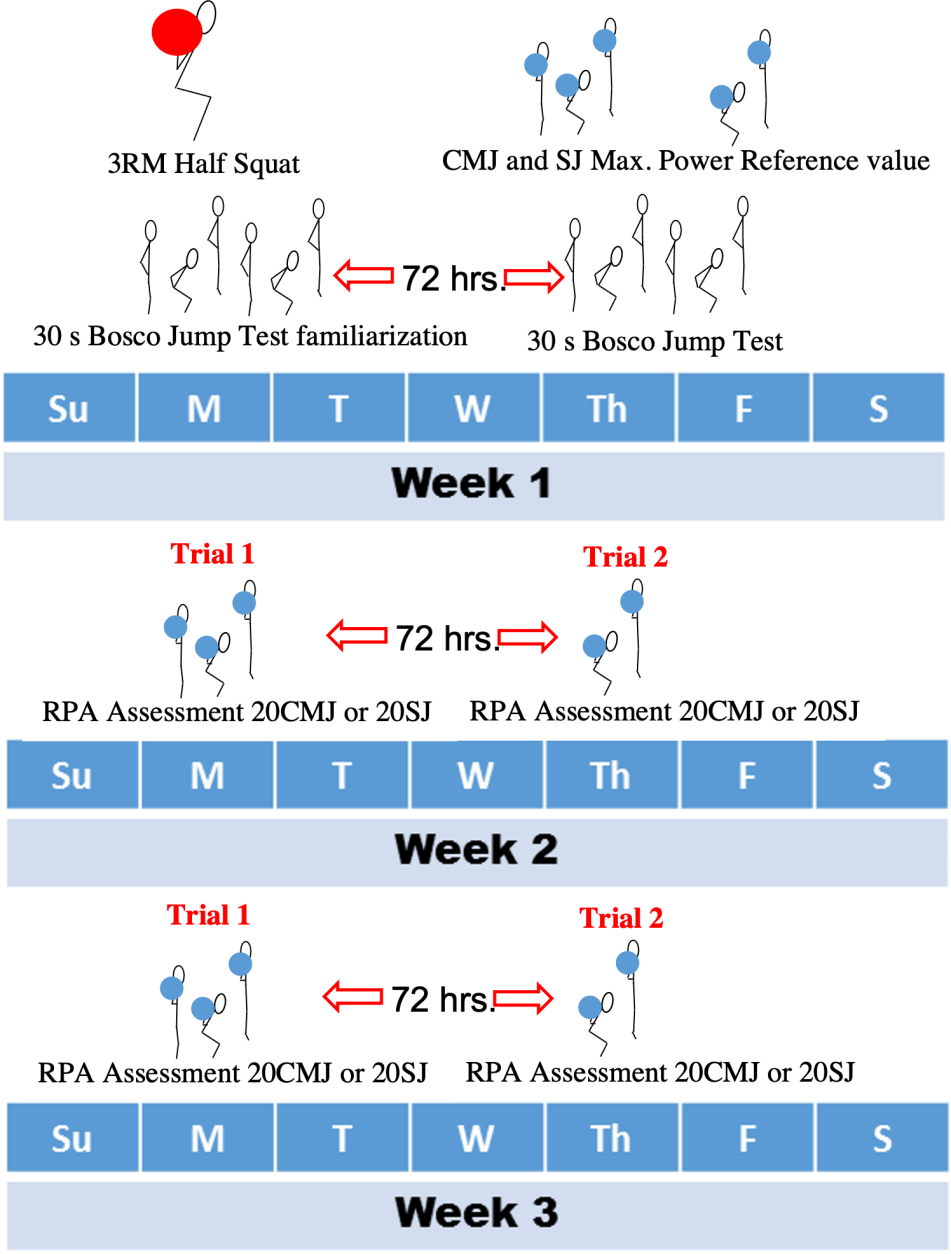
Data collection procedure. The 3 week data collection period, indicating week 1 for familiarisation, load quantification, reference value collection and the 30 second Bosco Jump Test assessment. Week 2 indicating the first trials of the Repeat Power Ability assessments with order of assessment, 20 repetitions of Squat Jumps or 20 repetitions of Countermovement Jumps, randomly assigned to each participant. Week 3 shown as a replication of week 2 for the repeat trial.

Participants were required to attend the strength and conditioning facility on six different occasions over a three-week period (see Fig. 1). The first session of week one was used to collect descriptive data and assess 3RM Half Squat (3RMHS) in order to estimate a 1RM Half Squat (1RMHS), which was used to calculate 30% of 1RMHS (30%HS) loads that were used for the RPA assessments. A percent 1RMHS load was chosen to stay consistent with regular strength training prescription guidelines and to allow RPA assessments to be able to cater for all strength levels, with jump loads based on individual capabilities. Along with 3RMHS testing, each participant performed a familiarisation trial with the 30BJT at the end of the first session. During session two, which was performed 72 h later, the 30BJT was assessed after establishing the maximal power reference value for both CMJ and SJ with the 30%HS load.

Weeks two and three were used for test-retest of the RPA protocols, where in a randomized order separated by 72 h, each participant performed either the 20SJ or the 20CMJ RPA assessment in different sessions. During week three, each of the RPA assessments were performed on the same corresponding day with the identical 48-hour period leading up to the assessment.

### Estimated one repetition maximum half squat

The 3RMHS assessment was conducted by an experienced Strength & Conditioning coach. The 3RMHS was performed with the use of a Smith machine (Fitech) and a full range of weight plates (Eleiko). A high bar placement was used, and squat descent was monitored to a depth where knee angle was 90°. Depth was established with the use of a goniometer and visually monitored. As a secondary precaution a thin rubber band was positioned so that each participant’s posterior thighs would contact the band when they reach the required depth (Thomasson & Comfort, 2012). Video recording from the sagittal plane was also used to analyse the depth of each squat attempt. The use of a Smith machine and the particular squat depth was required to match the equipment and depth of jump used in the RPA assessments.

After a standardised warm up consisting of 5 mins of stationary cycling at a rating of perceived exertion between 3-4 and a range of lower body and trunk dynamic flexibility exercises, a specific half squat warm up was conducted. After the standardized warm up, each participant, over three to four sub-maximal sets of one repetition, gradually built to their estimated 3RM load (Cronin & Hansen, 2005). They then attempted their estimated 3RM and with every successful attempt, and after three minutes of passive recovery, five kg was added until their 3RM had been reached (Cronin & Hansen, 2005). The last successful 3RM HS load lifted was converted into an estimated 1RM load using the average of seven different 1RM estimation formula’s (Epley, 1985; Lombardi, 1989; O’Connor, Simmons & O’Shea, 1989; Mayhew et al., 1992; Brzycki, 1993; Wathen, 1994; Lander, 1985). 30% 1RM HS loads were then extrapolated for the RPA assessments based on the mean estimated 1RM derived from these equations. As the validity of 1RM may be influenced by the exercise performed and population used in the respective studies, it was felt safer to use an average of these different 1RM estimation approaches.

### 30 second bosco jump test

The 30BJT consisted of thirty seconds of continuous compliant rebound jumps. Three minutes after completing the standardized warm up with the addition of a specific warm up consisting of three maximal CMJs, each participant stood on the force platform with hands on hips. On the command of “go” the participant performed thirty seconds worth of continuous compliant rebound jumps where upon landing they immediately sank to a visually and kinesthetically (with the use of a thin elastic band positioned to contact the posterior thigh) monitored depth of 90° knee flexion before jumping again. These jumps were performed in a continuous manner with no pause. Verbal encouragement and live auditory feedback of jump performance was provided by the investigators to assist in producing a maximal effort for each jump. Force-time data was collected for analysis with peak power and mean power for each jump along with the average of peak power and mean power for both fifteen second segments of the 30BJT calculated.

### Maximum jump assessment

Maximal jump performance using 30% 1RM HS loads was evaluated prior to RPA assessments in order to establish a “power standard” to be achieved within the first few jumps of the RPA assessments. Jumps were conducted with the use of a Smith machine with the fixed barbell held in the high bar position. The participants randomly initiated the jumps from either a standing position prior to a downward countermovement (CMJ) or from a static knee-hip flexed position (SJ). The depth for each jump type was again visually and kinesthetically monitored to 90° knee flexion. Participants were instructed to propel the system (combined body mass and barbell mass) as explosively as they could into the air, to reach a maximal jump height with each jump, in an attempt to maximize power output. Participants were also instructed to keep constant downward pressure on the bar in order for the barbell to remain in contact with their shoulders.

Three minutes after the standardised warm up, including three submaximal loaded jumps with the 30% 1RM HS load, each participant began the maximal jump assessment. A linear position transducer, sampling at the equivalent of 100 Hz, (Gymaware, Lisborne, Australia) was attached to the Smith machine bar and was used to calculate peak power output. The participants were required to perform a minimum of three jumps, with subsequent jumps required if there was an improvement of peak power output by a margin of ≤5% (Cormie, McBride & McCaulley, 2008). Three minutes rest was given between jumps and the jump registering the highest peak power was used as the “power standard” for the following RPA assessment.as used as the “power standard” for the following RPA assessment.

### Repeat power ability assessment

With each RPA assessment, 20 maximal repetitions of discontinuous jumps were performed 5 min after the standardized warm up including three maximal loaded jumps with the 30% 1RM HS load. During the RPA assessment each jump was performed every three seconds in time with the sound of a metronome, allowing limited inter-repetition recovery (∼1.5–2 s) whilst providing precise data collection points for each discrete jump. A Smith machine was used, with the fixed barbell held in the high bar position, with the depth of each jump monitored in two ways. The participants kinesthetically monitored each jump to a depth of 90° knee flexion (again with the use of the thin elastic band positioned to touch the posterior thigh at the required depth). The researcher, who was positioned perpendicular to the sagittal plane movement also visually monitored the depth of each jump, made a visual inspection of the athletes’ movement and any displacement of the elastic band was used to identify any obvious countermovement, with the trial ceasing if this did occur.

In the 20CMJ condition, upon each landing, participants assumed a full knee extension stance until the sound of the metronome, which signaled the immediate initiation for the countermovement of the next jump. Upon each landing in the 20SJ conditioning, each participant momentarily paused in an upright stance position before descending to the 90° knee bend position. The participants would then pause motionless for approximately 1 s awaiting the sound of the metronome, while eradicating any obvious countermovement or stretch shortening cycling motion before the jump. Whilst force-time data was collected on the force plates, instantaneous feedback of peak power output for each jump, derived from the linear position transducer, was visually displayed on a tablet screen placed in front of the participant and was also provided verbally after each jump by the investigators. Each participant was required to reach their “power standard” within the first three jumps of the RPA assessment to continue the assessment. The power standard was derived from the linear position transducer during the maximum jump assessments. Only force-time data collected on the force plates was further used to calculate peak and mean power for each jump.

### Data analysis

A force plate (ForceDecks; VALD Performance Systems, Brisbane, Australia) sampling at 1000 Hz was used to collect all jump data for the 20SJ, 20CMJ and the 30BJT. Force time data was analysed with customised MATLAB code using an extension on the methods used by Chavda et al. (2018). A second-order high-pass Butterworth filter with a cutoff frequency of 0.25 Hz was used to filter acceleration data to reduce drift caused by integrating derived acceleration data.

The initial quiet standing phase was manually identified for each data series. Pseudo-code of the propulsive phase derivation was applied as; toe-off (end of take-off phase) was identified as a point between the landing index of the previous jump (or quiet standing) and the index where the threshold of the net force remained below 50N for > 0.1s. (Chavda et al., 2018). Touch-down was identified as the index where net force returned to a value > 50N, post take-off (Chavda et al., 2018).

Peak landing phase power was identified as a value to identify other instances of the propulsion phase. Peak landing phase power was identified as the index of the maximum power occurring after touch-down (or quiet state) and before toe-off (of the next jump) with a minimum peak separation time of 700 ms (which reliably picked up every point correctly for the given dataset). Minimum power between peak propulsion and the peak power during the landing phase was used as another identification value.

To calculate propulsive power, first, acceleration was derived from the measured force by dividing by the subject’s mass at quiet. Acceleration was then filtered and integrated using a cumulative trapezoidal approximation to give velocity at each time instance. Next, power could be calculated by taking the previously obtained velocity at each time instance and multiplying it by the force at each corresponding instance. Finally, the propulsive phase was identified as the duration between the zero point crossing of the power trace (first occurrence after minimum power), and toe-off. Peak propulsive power could be determined as the peak power generated during the established propulsive phase, while mean propulsive power was calculated as the sum of the power samples during the propulsive phase divided by the number of propulsive phase power samples.

### Statistical analysis

Peak and mean power output measurements for each jump were used to calculate a fatigue index (FI%) using the following formula: Fatigue = ([highest power output − lowest power output]/highest power output × 100). A percent decrement score (PD%) was calculated using the following formula: Fatigue = 100 × [total jump power/ideal jump power] − 100. Average power (avg) was also calculated using the formula; total jump power/number of jumps (Patterson, Platzer & Raschner, 2019; Girard, Mendez-Villanueva & Bishop, 2011; Glaister et al., 2004).

FI%, PD% and Avg with the first and final jumps removed were also calculated. Removing the first and final jumps for power decline calculations was justified due to the greater variability of these than the remaining 18 jumps.

Peak and mean power measures recorded for each jump and the average peak and mean power measures for both 15 s segments during the 30BJT were used to establish FI%, PD% and Avg scores. A summary of these variables and their abbreviations is provided in Table 1. Statistical analysis was conducted using JASP online statistical package, Microsoft Excel and Hopkin “Analysis of Reliability with a Spreadsheet. (beta version, October 21, 2022).

**Table 1:** Abbreviation of variables.

Variable	Abbreviation
Average peak power output for 20 Countermovement jumps	AvgCMJ_peak_
Average peak power output for 18 Countermovement jumps	AvgCMJ_peak18_
Peak power output Fatigue Index % for 20 Countermovement jumps	FI%CMJ_peak_
Peak power output Fatigue Index % for 18 Countermovement jumps	FI%CMJ_peak18_
Peak power output Percent Decrement % for 20 Countermovement jumps	PD%CMJ_peak_
Peak power output Percent Decrement % for 18 Countermovement jumps	PD%CMJ_mean18_
Average mean power output for 20 Countermovement jumps	AvgCMJ_mean_
Average mean power output for 18 Countermovement jumps	AvgCMJ_mean18_
Mean power output Fatigue Index % for 20 Countermovement jumps	FI%CMJ_mean_
Mean power output Fatigue Index % for 18 Countermovement jumps	FI%CMJ_mean18_
Mean power output Percent Decrement % for 20 Countermovement jumps	PD%CMJ_mean_
Mean power output Percent Decrement % for 18 Countermovement jumps	PD%CMJ_mean18_
Average peak power output for 20 Squat jumps	AvgSJ_peak_
Average peak power output for 18 Squat jumps	AvgSJ_peak18_
Peak power output Fatigue Index % for 20 Squat jumps	FI%SJ_peak_
Peak power output Fatigue Index % for 18 Squat jumps	FI%SJ_peak18_
Peak power output Percent Decrement % for 20 Squat jumps	PD%SJ_peak_
Peak power output Percent Decrement % for 18 Squat jumps	PD%SJ_mean18_
Average mean power output for 20 Squat jumps	AvgSJ_mean_
Average mean power output for 18 Squat jumps	AvgSJ_mean18_
Mean power output Fatigue Index % for 20 Squat jumps	FI%SJ_mean_
Mean power output Fatigue Index % for 18 Squat jumps	FI%SJ_mean18_
Mean power output Percent Decrement % for 20 Squat jumps	PD%SJ_mean_
Mean power output Percent Decrement % for 18 Squat jumps	PD%SJ_mean18_

Within-subject variation between corresponding RPA assessments, using each measure of power decline, were derived using intraclass correlations coefficients (ICC) and coefficient of variation (CV). ICC reliability interpretation thresholds were based off lower bound 95% confidence intervals ranging from poor (<0.5), moderate (0.5–0.75), good (0,75–0.9) and excellent (>0.9) reliability (Koo & Li, 2016). A CV of ≤ 10% was considered good absolute reliability (Hopkins, 2000). A paired samples *t*-test was used to determine significant differences between test occasions, with a *p*-value set at <0.05. A Hedge’s g effect size was used to determine the magnitude of differences between the test occasions with interpretation thresholds set as; trivial (<0.2), small (0.21–0.59), moderate (0.6–1.19), large (1.2–1.99) and very large (>2.00) (Hopkins, 2010).

Pearson’s correlation coefficients were used to identify associations between all RPA power decline measures and the 30BJT. The thresholds to interpret the magnitude of correlation were trivial (<0.1), small (0.11–0.3), moderate (0.31–0.5), large (0.51–0.7), very large (0.71–0.9) and almost perfect (0.91–1.0) (Hopkins et al., 2009). Validity limits of agreement were compared between all fatigue measures in each RPA condition and the corresponding power decline measures in the 30BJT with validity set at ≥ 0.9. A Bland-Altman plot was further used to compare the level of agreement between the most reliable measurement of RPA and the corresponding 30BJT variable.

## Results

**Figure 2:**
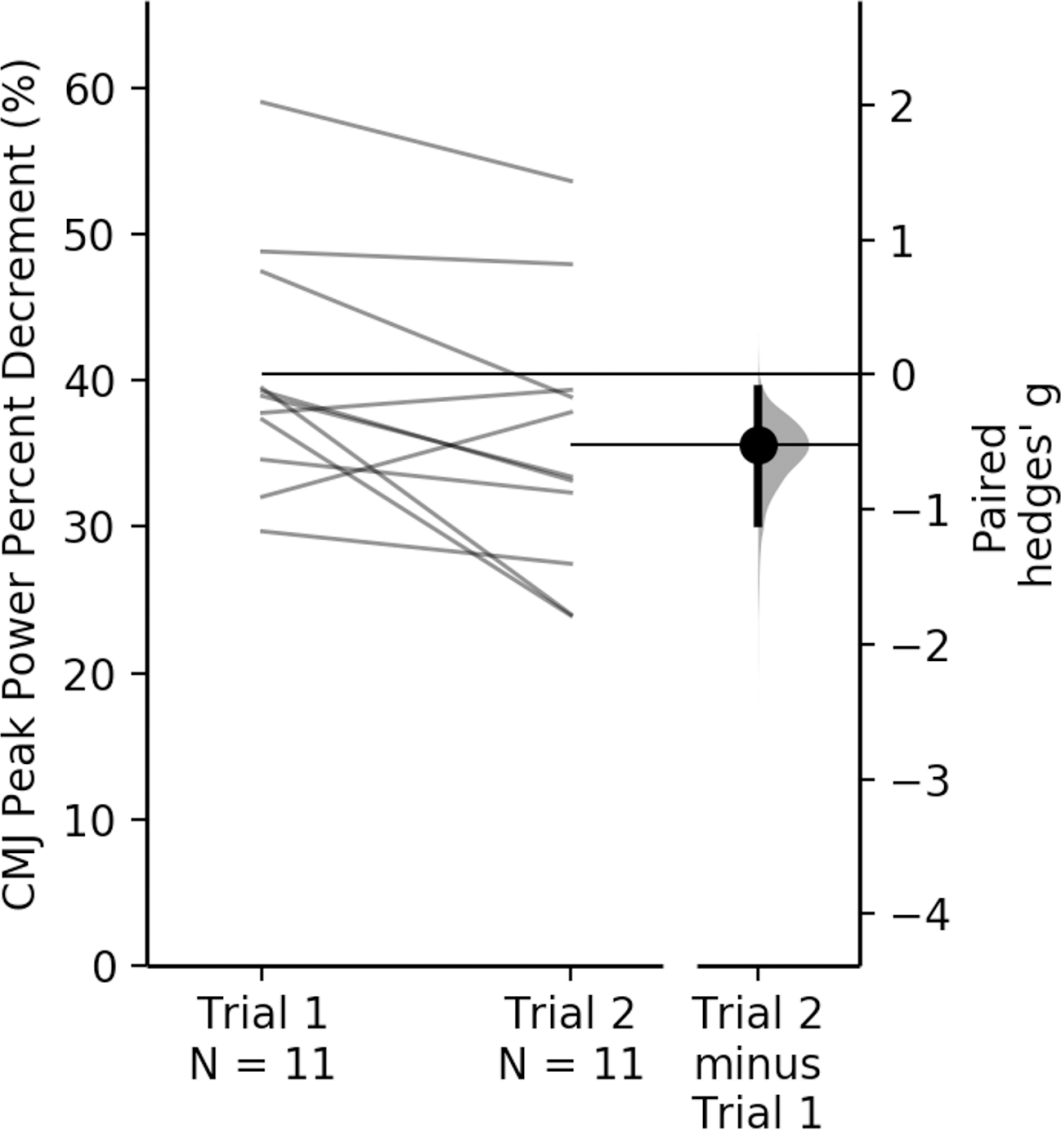
Between trial comparison of CMJ peak power output percent decrement with the first and final repetitions removed. Each data point in each trial represents one participants percent decrement (%) peak power output score with the first and last repetition removed. Each individual line connects the participant between their first and second trial.

AvgCMJ_peak_ and AvgSJ_peak_ had good intertest reliability (CV = 2.5–3.8; ICC = 0.94–0.96 [CI [0.83–0.991]]). AvgCMJ_mean_ had better reliability than AvgSJ_mean_ (CV = 3; ICC = 0.96–0.96 [CI [0.884–0.991]] *vs* CV = 7.2–7.4; ICC—0.841–0.846 [CI [0.534–0.955]]). PD%CMJ_peak18_ (Fig. 2) was the most reliable measurement of power output decline (CV = 4.9; ICC = 0.85 [CI [0.62–0.964]]).

**Figure 3:**
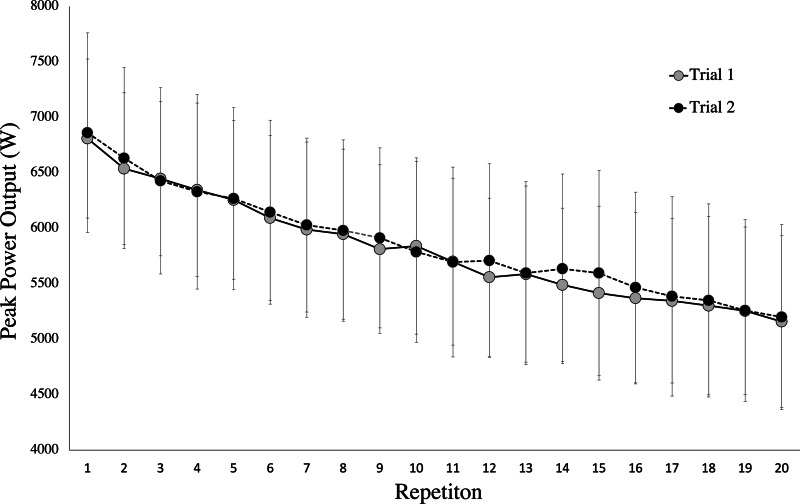
Peak power output for all participants for each repetition for both trials of the CMJ20 protocol. Each data point indicates the mean and and standard deviation for peak power output for each repetition of each trial for the CMJ20 protocol.

There were no significant differences found between the two testing occasions for all variables (*p* = 0.105–0.858) except for PD%SJ_peak18_ and PD%CMJ_mean18_ (*p* = 0.029 and *p* = 0.045 respectively). In establishing the magnitude of difference between trials only small differences were observed (Hedges g effect = 0.517) All participants displayed similar peak power output for each trial (Fig. 3) with a mean first repetition of 6834.8 ± 808.2 W and a 20th repetition of 5178.9 ± 804.5 W for both trials. The mean decrease between 1st and 2nd repetitions was 250.2 ± 57.7 W and the mean decrease from 19th to 20th repetitions was 77.6 ± 16.3 W. The first and/or last repetition, in the present study have high variability between trials. Therefore, when removing them from the calculation the CV for PD%CMJ_peak_ improved from 20.5% to 4.9% for PD%CMJ_peak18_.

**Figure 4:**
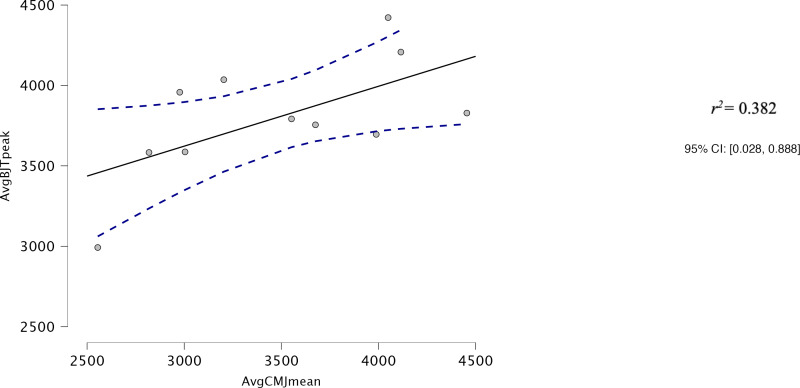
Correlation for Avg mean power output for CMJ20 and the Avg peak power output of the 30BJT.

**Figure 5:**
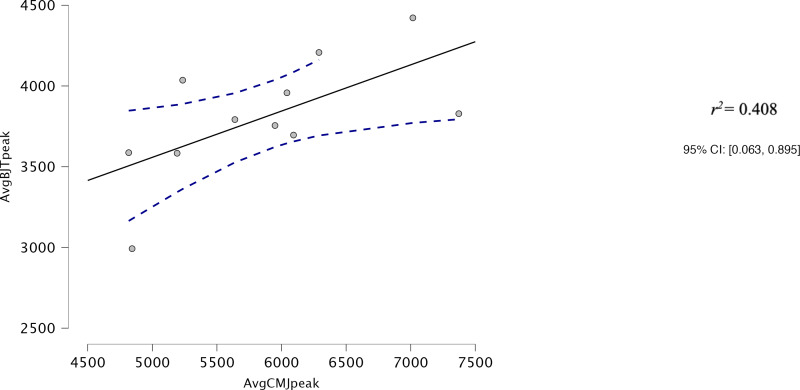
Correlation for Avg peak power output for CMJ20 and the Avg peak power output of the 30BJT.

**Figure 6:**
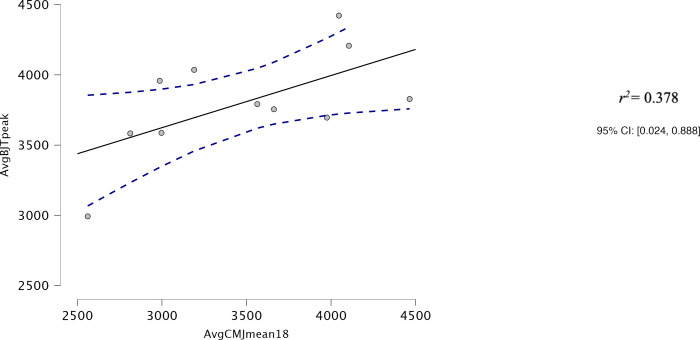
Correlation for Avg mean power output of CMJ20, with the first and last repetitions removed, and the Avg peak power output of the 30BJT.

**Figure 7:**
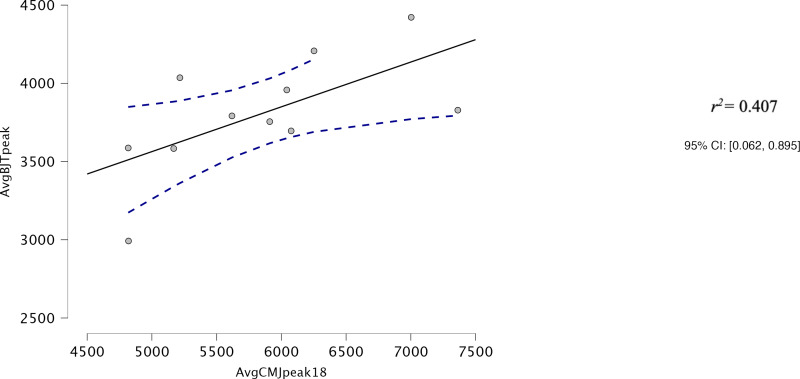
Correlation for Avg peak power output of the CMJ20, with the first and last repetitions removed, and the Avg peak power output of the 30BJT.

**Figure 8:**
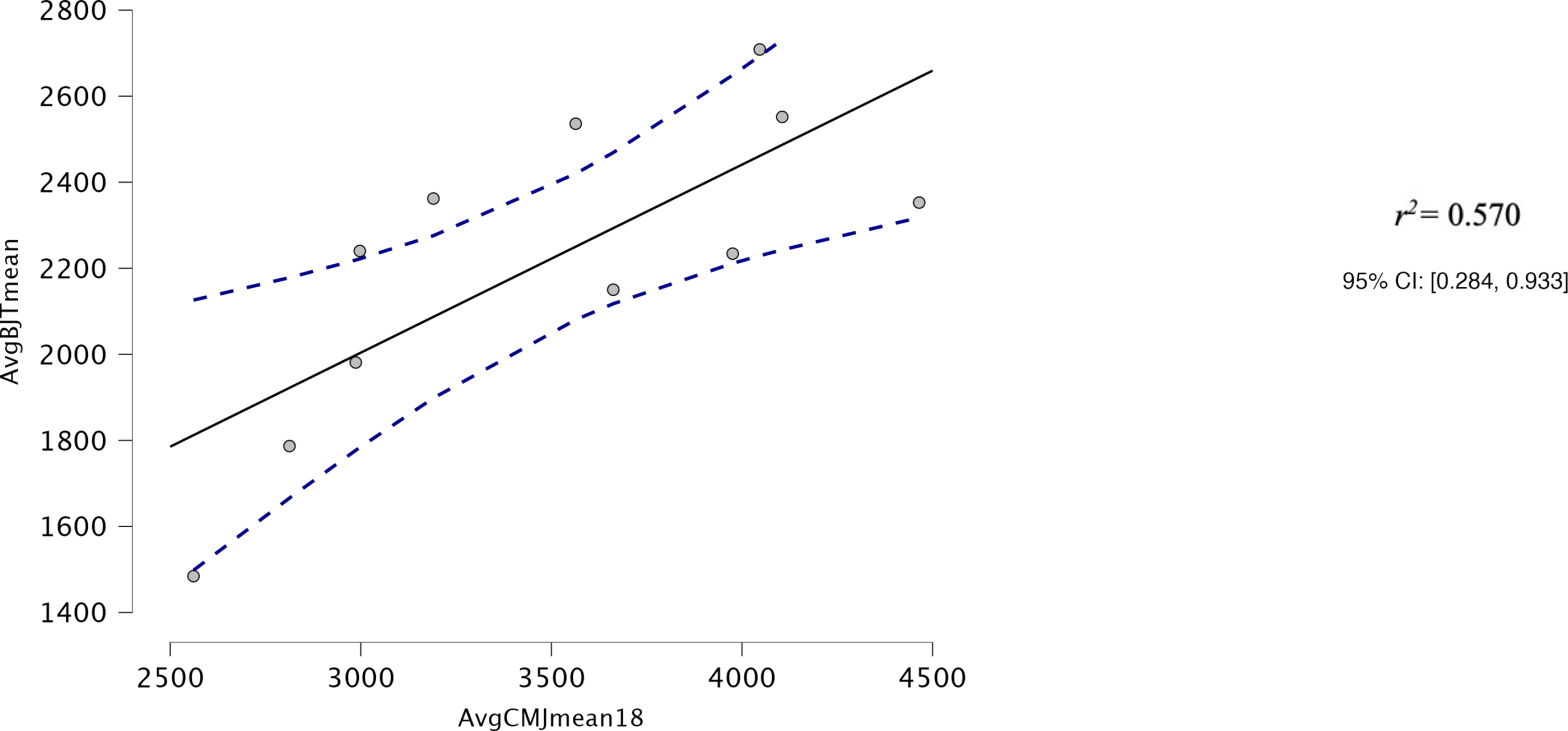
Correlation for Avg mean power output of the CMJ20, with the first and last repetitions removed, and the Avg mean power output of the 30BJT.

**Figure 9:**
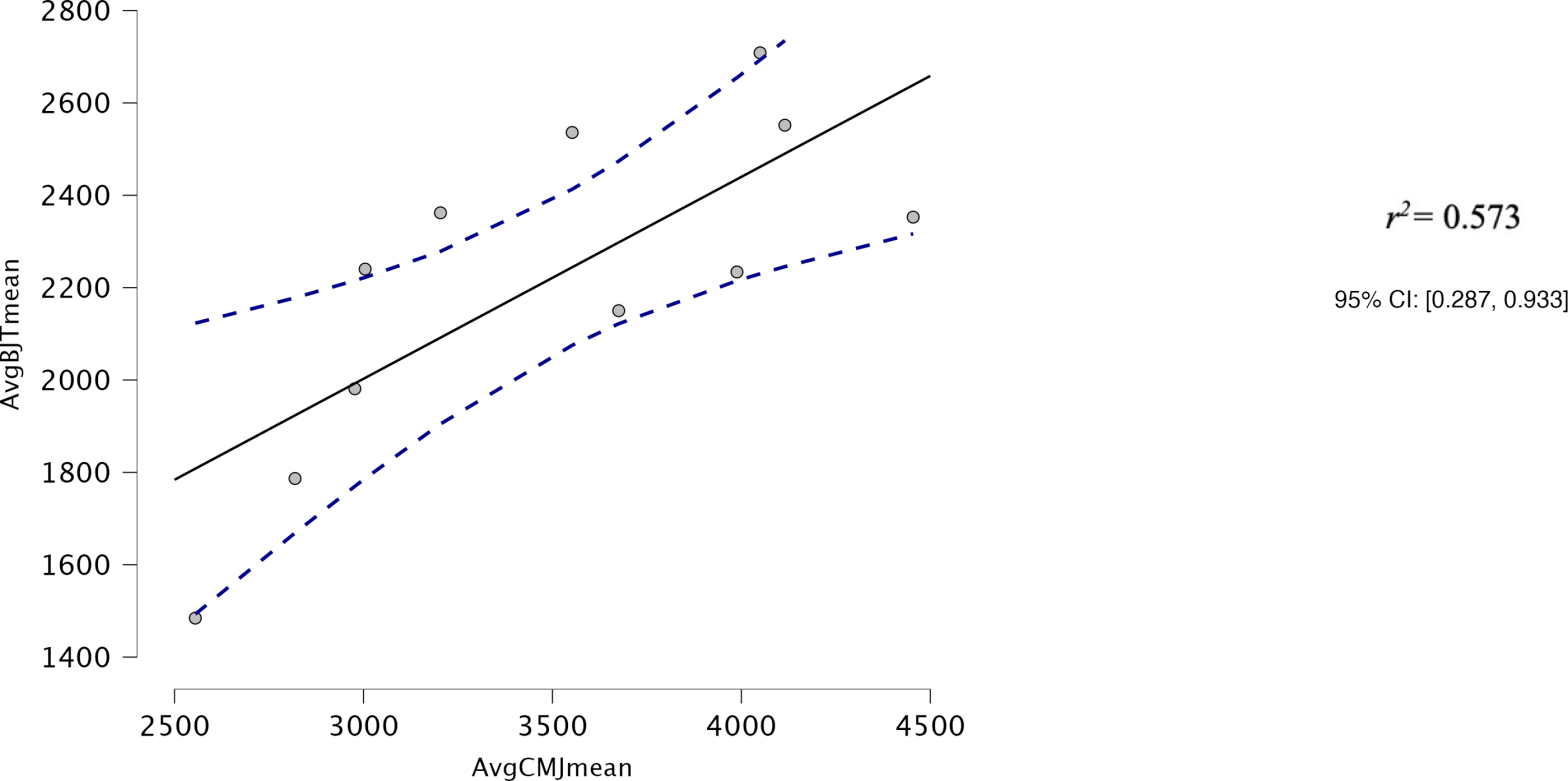
Correlation for Avg mean power output of the CMJ20, with the first and last repetitions removed, and Avg mean power output of the 30BJT.

Both AvgCMJ_mean_ and AvgCMJ_peak_ had moderate correlations with Avg30BJT_peak_ (*r* = 0.618, *p* = 0.043 and *r* = 0.639, *p* = 0.34 respectively) (Figs. 4 and 5). AvgCMJ_mean18_ and AvgCMJ_peak18_ also had moderate correlations with Avg30BJT_peak_ (*r* = 0.615, *p* = 0.044 and *r* = 0.638, *p* = 0.035 respectively) (Figs. 6 and 7). AvgCMJ_mean_ and AvgCMJ_mean18_ had strong correlations with Avg30BJT_mean_ (*r* = 0.757, *p* = 0.007 and *r* = 0.755, *p* = 0.007) (Figs. 8 and 9).

**Figure 10:**
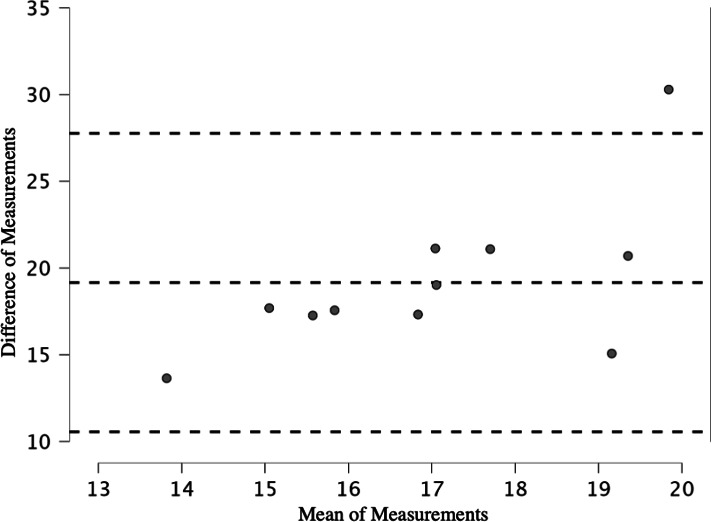
Limits of agreement for percent decrement of CMJ20 peak power output, with the first and last repetitions removed, and percent decrement of peak power output for the 30BJT.

No RPA measurements of power decline were significantly related to 30BJT measurements of power decline. The limits of agreement between PD%CMJ_peak18_ and percent decrement of 30BJT peak power (Fig. 10) were poor (bias = 19.164, limits of agreement = 10.56–27.768).

## Discussion

The results from this study show that an RPA assessment consisting of either 20 repetitions of CMJs or SJs with a 30% 1RM load can be a reliable assessment for outcomes reflecting average power output. Specifically, both the average peak and average mean power output for the 20CMJ had good reliability, whereas only the average peak power output in the 20SJ had good reliability. When removing the first and last jumps from the calculation, the 20CMJ PD% score proved to be the most reliable measure of power decline and is therefore the recommended measurement of RPA. Although there were large to very large associations found between Avg CMJ peak and mean power output with 30BJT peak and mean power output, measures of power output decline had only trivial to moderate associations and with no validity established between the assessments.

Relative test re-test reliability of the 20CMJ using the PD%CMJ_peak18_ had better relative test re-test reliability than the Kansas Squat Test in the assessment of power decline (ICCs = 0.875 *versus* 0.754 respectively) (Fry et al., 2014). The Kansas Squat Test is an RPA assessment utilising a traditional strength training lift consisting of 15 repetitions of back squats executed at maximal velocity. Each repetition of the Kansas Squat Test was performed every 6 s, with a system mass back squat load of 70% 1RM. To minimise variability in the Kansas Squat Test, the time between repetitions, depth of squat, vertical tracking of the barbell and technical execution of the squat, in not allowing ankle plantar flexion at the end of the ascent, were all controlled. In the current study, jump depth, timing of jump and vertical tracking of the barbell were also controlled, however, the ballistic movement of the jump allowed for a continuation of momentum and greater acceleration throughout the range of movement. Allowing for the continuation of momentum may have contributed to the 20CMJ being a more reliable assessment of RPA than the Kansas Squat Test. With the ability to accelerate continuously throughout the movement, the 20CMJ may also more closely replicate sporting movements than the Kansas Squat Test (Newton et al., 1996).

Although absolute reliability was not assessed in the Kansas Squat Test, Alemany et al. (2005) did examine absolute reliability in their RPA assessment consisting of 30 loaded CMJs with a 30% 1RM back squat load. The loaded CMJs were performed consecutively with no pause between jumps and with self-selected depth. In comparing both AvgCMJ_peak_ and AvgCMJ_mean_ with the average peak and mean power output in the Alemany et al. (2005) assessment, the 20CMJ is a comparable or slightly more reliable RPA assessment (CVs = 2.5–3% *vs* 3.2–4.4%, respectively). In visually examining a representative fatigue curve illustrated by Alemany et al. (2005), it appears that their use of 30 continuous jumps caused a more substantial decline in power output than the present study (∼50% decline from first to last repetition *vs* ∼25% decline from first to last repetition, respectively).

In both the current study and the work of Alemany et al. (2005), CMJ peak power output was found to be more reliable than mean power output. Mean power output represents the rate of mechanical work done over the whole propulsive phase of the jump as opposed to peak power output which represents a specific sample period of the highest rate of mechanical work during the propulsive phase (Gathercole et al., 2015). In RPA assessments, a longer sampling period across the CMJ propulsive phase seems to introduce greater variability and therefore less reliability between trials. Much of the variability captured in the longer sampling period may reflect the greater ability to capture changing movement strategies used to try to maintain power output. This variability in mean power output seems to be consistent across both loaded and unloaded CMJ conditions (García-Ramos et al., 2016; Hori et al., 2009; Cormack et al., 2008). Peak power output is therefore the recommended metric for assessing RPA.

The current study also sought to examine whether jump type may influence reliability, and it appears to be the only known study to use SJs in an RPA assessment, which might be particularly important for athletes needing to repeatedly perform ballistic concentric only actions. However, it has also been proposed that in a SJ, it can be difficult to maintain a stable and static period before the initiation of the propulsive phase of the jump (García-Ramos, Pérez-Castilla & Jaric, 2021; Pérez-Castilla et al., 2021). In the 20SJ, where fatigue induced declines in power output even with live feedback of power output, it is likely that technical strategies to maintain power output could be used more readily under these conditions. One such strategy might be performing a small countermovement to potentiate the propulsive phase of the jump (Dugan et al., 2004). Holding a static position under increasing levels of fatigue along with the potential use of a countermovement would introduce a higher level of variability in the 20SJ as opposed to the 20CMJ and may be the reason why no RPA studies to date have used SJs. Despite this potential variability, the 20SJ did show good reliability for AvgSJ_peak_ and AvgSJ_peak18_.

**Figure 11:**
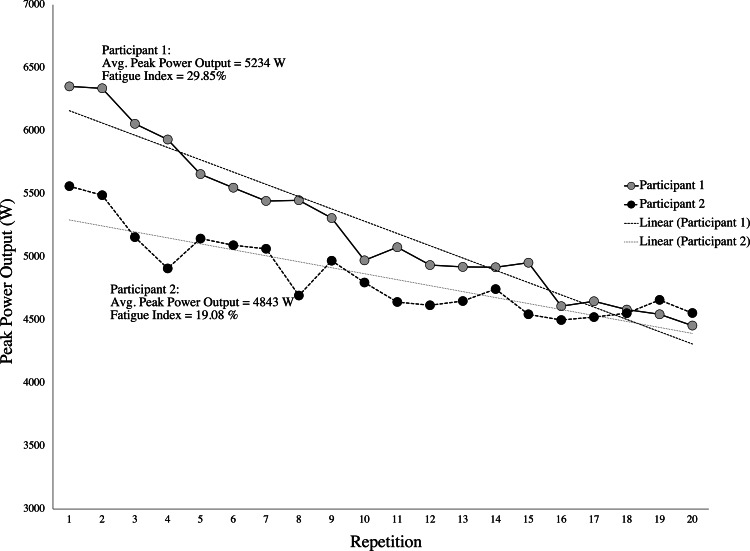
Example of peak power output for two participants. Data points and line of best fit for participant 1 showing a higher average peak power output with a large fatigue index % in comparison to participant 2 with lower average peak power output and a smaller fatigue index %.

In general, across both the 20CMJ and 20SJ, the Avg power output scores are the most reliable calculation identified. Avg power output scores could be used longitudinally for an individual athlete to understand their ability to maintain power output. However, an average power output score does not provide a clear measure of power decline and can often be heavily influenced by maximal power output (Douglas, Ross & Martin, 2021). For example, due to a higher starting threshold an athlete with a high maximal power output but high levels of power decline, can have comparatively higher overall Avg power output than an athlete with a lower peak power output threshold and power decline (see example of this between two different participants in Fig. 11). For comparisons of RPA to be made between athletes, it is recommended that a power decline score be used to describe an athlete’s ability more precisely to repeatedly produce maximal muscular efforts against external resistances.

In the current study, a FI% (Fatigue = ([highest power output − lowest power output]/highest power output × 100)) and a PD% (Fatigue = 100 × [total jump power/ideal jump power] − 100) were used to quantify power output decline. When calculating the FI% and PD%, neither of the RPA assessments were found to be reliable when all 20 repetitions were incorporated into the calculation. However, when the first and last repetition were removed from the calculations, reliability significantly improved with PD% showing the best reliability (CV = 4.9%). PD% has previously been shown to be the most valid and reliable method of quantifying fatigue where each repetition is factored into the overall result rather than a finite representation (Glaister et al., 2004). Again, the first and/or last repetition, in the present study have high variability between trials. Therefore, when removing them from the calculation, the CV for PD%CMJ_peak_ improved from 20.5% to 4.9% for PD%CMJ_peak18_. It is suggested that the knowledge of repetition number in repeated maximal efforts tasks can lead to the use of significant pacing strategies (Billaut et al., 2011). The first and last repetition of the CMJ20 seems to be highly susceptible to some form of pacing and therefore the removal of these repetitions from the PD%CMJ_peak_ calculation appears to better represent RPA and substantially improve absolute reliability.

None of the RPA power decline assessments were related to the 30BJT power decline assessments. The continuous nature of the 30BJT *versus* the brief between jump rest periods provided in the 20CMJ and 20SJ and the higher number of jumps performed in the 30BJT *versus* the 20CMJ and 20SJ may have contributed to this. This finding may suggest that the addition of load in an RPA assessment presents a substantially different physical challenge that alters the power decline profile observed in an RPA assessment with no external load. However, despite the reduced number of jumps and the brief recovery between jumps in the 20CMJ and 20SJ, the addition of load seems to have provided a higher stress to the neuromuscular and cardiorespiratory systems and in turn altered the power decline profile more readily then in the 30BJT. This is evident for example when comparing the mean and standard deviations in PD% between PD%CMJ_peak18_ and PD%30BJT_peak_ (26.61 ± 3.71% *versus* 7.44 ± 1.77% respectively). It appears that PD%CMJ_peak18_ and the PD%BJT30_peak_ are both unique assessments and measurement indices. The poor level of agreement between these two assessments is further evidence of the disparity between these two measures and further confirmation that validity is not established between these assessments.

When comparing Avg mean and peak power output for the 20CMJ against the Avg mean and peak power output for the 30BJT, large to very large relationships were observed. However, as Avg measures can be substantially influenced by maximal power output, the relationship is likely more indicative of the association between maximal power output in a relative 1RM loaded jump condition *versus* a jump condition with no external load.

This is the first study to examine jump type, measurement indices and power decline calculations to determine the most reliable ballistic assessment of RPA in well trained athletes. A limitation to this study may be the calculation of power decline for the 30BJT assessment. In the current study we followed the recommendations provided in the original research by Bosco et al. (1983) using the 60BJT, where the average of power output for the first 15 s was compared to the average of the final 15 s of the 30BJT. Perhaps factoring in each individual jump, as done in the CMJ20 and the SJ20 power decline calculations, may have provided a better comparison between the assessments.

## Conclusions

We hypothesized that the PD%CMJ would be the most reliable RPA assessment, however removing the first and last repetition made PD%CMJ_peak18_ the most reliable measure of RPA power output decline. As also hypothesized, no RPA assessment and measurement was valid with the 30BJT. In comparing mean power output and peak power output for 20SJ and 20CMJ with various methods of quantifying power output decline, we found the PD% score of peak power output for CMJ20, with the first and last jumps removed, to be the most reliable assessment of RPA. The addition of load, the reduced number of jumps and the discontinuous nature of jumps in the CMJ20 appear to have a differing effect on the rate and magnitude of power decline seen in 30BJT. The 20CMJ assessment now provides a reliable ballistic RPA assessment, that with the addition of load, may be more applicable to certain sporting movements and actions whilst being simple to administer and conduct in the field. Future research should look to further explain and define CMJ20 by identifying related physical qualities and relationships with aspects of competition performance.

## Additional Information and Declarations

### Data Availability

The following information was supplied regarding data availability: All raw force-time data for all jumps and MATLAB code used to analyse the force-time data are available in figshare: Natera, Alex (2023): Loaded Countermovement Jump 1x20 Data. figshare. Dataset. https://doi.org/10.6084/m9.figshare.23167661.v1. Natera, Alex (2023): Loaded Squat Jump 1x20 Data. figshare. Dataset. https://doi.org/10.6084/m9.figshare.23168279.v1. Natera, Alex (2023): 30s Bosco Jump Data. figshare. Dataset. https://doi.org/10.6084/m9.figshare.23166755.v1. Natera, Alex (2023): Matlab Code - all jump data. figshare. Software. https://doi.org/10.6084/m9.figshare.23168339.v1.
